# Tracking Therapy Response in Glioblastoma Using 1D Convolutional Neural Networks

**DOI:** 10.3390/cancers15154002

**Published:** 2023-08-07

**Authors:** Sandra Ortega-Martorell, Ivan Olier, Orlando Hernandez, Paula D. Restrepo-Galvis, Ryan A. A. Bellfield, Ana Paula Candiota

**Affiliations:** 1 Data Science Research Centre, Liverpool John Moores University, Liverpool L3 3AF, UK; i.a.oliercaparroso@ljmu.ac.uk (I.O.); r.a.bellfield@2019.ljmu.ac.uk (R.A.A.B.); 2 Escuela Colombiana de Ingeniería Julio Garavito, Bogota 111166, Colombia; orlando.hernandez@mail.escuelaing.edu.co (O.H.); paula.restrepo-g@mail.escuelaing.edu.co (P.D.R.-G.); 3 Centro de Investigación Biomédica en Red: Bioingeniería, Biomateriales y Nanomedicina, 08193 Cerdanyola del Vallès, Spain; 4 Departament de Bioquímica i Biologia Molecular, Facultat de Biociències, Universitat Autònoma de Barcelona, 08193 Cerdanyola del Vallès, Spain

**Keywords:** therapy response, glioblastoma, temozolomide, preclinical models, magnetic resonance spectroscopy, class activation mapping, Grad-CAM, convolutional neural networks, deep learning

## Abstract

**Simple Summary:**

Glioblastoma (GB) is a malignant brain tumour with no cure, even after the best treatment. The evaluation of a therapy response is usually based on magnetic resonance imaging (MRI), but it lacks precision in early stages, and doctors must wait several weeks until they are confident information is produced, facing an uncertain time window. Magnetic resonance spectroscopy (MRS/MRSI) can provide additional information about tumours and their environment but is not widely used in clinical settings since the spectroscopy format is not standardised as MRI is, and doctors are not familiarised with outputs/interpretation. This study aims to improve the assessment of the treatment response in GB using MRSI data and machine learning, including state-of-the-art one-dimensional convolutional neural networks. Preclinical (murine) GB data were used for developing models that successfully identified tumour regions regarding their response to treatment (or the lack thereof). These models were accurate and outperformed previous methods, potentially providing new opportunities for GB patient management.

**Abstract:**

Background: Glioblastoma (GB) is a malignant brain tumour that is challenging to treat, often relapsing even after aggressive therapy. Evaluating therapy response relies on magnetic resonance imaging (MRI) following the Response Assessment in Neuro-Oncology (RANO) criteria. However, early assessment is hindered by phenomena such as pseudoprogression and pseudoresponse. Magnetic resonance spectroscopy (MRS/MRSI) provides metabolomics information but is underutilised due to a lack of familiarity and standardisation. Methods: This study explores the potential of spectroscopic imaging (MRSI) in combination with several machine learning approaches, including one-dimensional convolutional neural networks (1D-CNNs), to improve therapy response assessment. Preclinical GB (GL261-bearing mice) were studied for method optimisation and validation. Results: The proposed 1D-CNN models successfully identify different regions of tumours sampled by MRSI, i.e., normal brain (N), control/unresponsive tumour (T), and tumour responding to treatment (R). Class activation maps using Grad-CAM enabled the study of the key areas relevant to the models, providing model explainability. The generated colour-coded maps showing the N, T and R regions were highly accurate (according to Dice scores) when compared against ground truth and outperformed our previous method. Conclusions: The proposed methodology may provide new and better opportunities for therapy response assessment, potentially providing earlier hints of tumour relapsing stages.

## 1. Introduction

Glioblastoma (GB) is an aggressive and highly malignant brain tumour that is notoriously difficult to treat. Standard treatment typically involves surgical resection, followed by radiation therapy and chemotherapy with temozolomide (TMZ), known as the Stupp protocol [1], with an average survival of 12–15 months after diagnosis. Despite advancements involving novel therapy research, GB often recurs, and the prognosis remains poor.

Therapy response follow-up in GB is paramount in improving patient outcomes and guiding treatment decisions, and it is mostly based on magnetic resonance imaging (MRI). The most widely utilised criteria are the Response Assessment in Neuro-Oncology (RANO) criteria [2], which provide guidelines for evaluating treatment responses in brain tumours, including GB. The criteria consider various factors, including changes in the tumour size, enhancement pattern and clinical status, to categorise treatment response into specific categories such as complete response, partial response, stable disease or progressive disease. 

However, the early assessment of therapy efficacy is usually hampered by phenomena such as pseudoprogression and pseudoresponse [3]. Patients and doctors may have to wait several weeks in order to confirm whether a therapeutic approach might be maintained or, contrarily, whether it should be halted and changed to a second-line therapeutic agent due to a lack of a response. Precious time is wasted, contributing to the poor outcome in GB-afflicted patients. 

Magnetic resonance (MR) can also provide metabolomics information about the tumour and its microenvironment, namely, MR spectroscopy or spectroscopic imaging (MRS/MRSI), which are known to provide earlier information about the response to therapy and preclinical and clinical settings [4,5]. Nevertheless, spectroscopic approaches are currently underused in clinical settings, since doctors are not fully familiarised with the output of the acquisition, as well as its processing, postprocessing, quantitation and interpretation. The lack of standardisation in file formats and formal studies of its added value (with some exceptions, such as [6]) do not contribute to the implementation of MR spectroscopic approaches in clinical settings.

Our groups have been working in the preclinical scenario in order to explore, interpret, analyse and validate the potential of the spectroscopic approaches, especially spectroscopic imaging (MRSI), in the early assessment of therapy responses [7,8,9]. Also, for several years now, machine learning (ML) and deep learning (DL) methods have been consistently shown to be able to help with tasks such as brain tumour detection [10,11,12], diagnosis and grading [13,14,15], classification [16,17,18,19], segmentation of the affected/tumour area [20,21,22] and therapy response prediction to distinguish post-treatment effects and tumour progression [23,24,25,26,27], among others. Hence, we firmly believe that ML and DL methods applied to such metabolomic datasets using whole pattern inputs may help to unravel its potential in a way that would not be possible with a single metabolite or ratio quantitation.

There is currently a strong recommendation regarding applying advanced MRI techniques for treatment response assessment in gliomas [28], including spectroscopy, which might also require the use of advanced ML techniques to properly analyse, integrate and take advantage of such complex information, but there are only a few reported studies including CNN in MR spectroscopic studies related to glioma or glioma therapy follow-up. Specifically, the use of convoluted neural networks in glioblastoma studies and incorporating spectroscopic-related data (such as MRSI, which contains rich metabolomics information) is not widely reported in the literature. An example of these studies is Acquarelli et al. [29] for grading gliomas (grades 3 and 4) and identifying Alzheimer’s disease patients using MRSI and MRI data, identifying spectral regions most important in the diagnosis process. The application of neural networks to single-voxel spectroscopic data for tumour grading has been also reported [30]. The use of such approaches with MR Imaging data has been described to be useful for high-grade glioma prognosis [31], diagnosing brain tumours [32,33], grading and subtyping glioma [34], planning radiation therapy in brain tumours [35] or tumour segmentation [36]. Overall, it seems that our work could contribute, through the analysis of preclinical glioblastoma MRSI data, to unravelling the potential of CNN for therapy response assessment using metabolomics for this purpose.

In order to perform detailed studies for MRSI acquisition, optimisation and the cellular/molecular validation of our findings, we rely on the GL261 GB preclinical model, which is able to mimic human GB in features such as invasiveness, proliferation and palisade cellular structures, and which presents a transient/sustained response to TMZ, depending on the therapeutic schedule used. Then, in our previous work [5], we developed a semi-supervised approach which uses non-negative matrix factorisation (following the method proposed in [37]) in a defined cohort and defined paradigmatic spectra for three conditions, namely: normal brain parenchyma, untreated or unresponsive GB and GB treated–responding to therapy. The semi-supervised method could be applied to new individuals [9] and was able to correctly distinguish responses not only with TMZ but also with cyclophosphamide [38] and immune checkpoint inhibitors such as anti-PD1 alone or in TMZ combination [39]. However, there are still some issues with room for improvement, such as the appearance of a ‘responding’ pattern when tumours are in the late relapsing stage or getting earlier hints on tumour relapsing stages. 

In this paper, we aim to improve our previous work’s performance [5]. For this, we propose the use of convolutional neural networks (CNNs) [40], which are also tested and compared against four other widely used machine learning algorithms.

## 2. Materials and Methods

### 2.1. MRI/MRSI Data Used in This Study

#### 2.1.1. Preclinical Model Generation and Treatment Administration

Mice were obtained from Charles River Laboratories (France) and housed at the animal facility of the Universitat Autònoma de Barcelona (Servei d’Estabulari, https://sct.uab.cat/estabulari/content/presentaci%C3%B3.html, accessed on 4 August 2023). Tumours were induced by an intracranial stereotactic injection of 10^5^ GL261 glioma cells in the caudate nucleus, as previously described by us in [41]. TMZ was administered intragastrically to tumour-bearing mice in three cycles of 5, 2 and 2 days interleaved with 3-day intervals. The periods of administration were days 11 to 15, 19–20 and 24–25 post-implantation (considering ‘day 0’ as the tumour generation day) at a dose of 60 mg/kg per day of treatment. For this purpose, the stock TMZ solution was diluted in the administration vehicle (DMSO 10% in saline), and the volume administered was 200 μL per animal (taking 20 g per animal as the mean weight). Mice were coded as CXXXX, XXXX being a correlative number for their identification used in our group.

#### 2.1.2. MR Data Acquisition

MR studies were performed at the joint nuclear MR facility of UAB and CIBER-BBN, Unit 25 of NANBIOSIS (https://www.nanbiosis.es/portfolio/u25-nmr-biomedical-application-i/, accessed on 4 August 2023), with a 7 Tesla horizontal magnet (BioSpec 70/30, Bruker BioSpin, Ettlingen, Germany). The MRI and MRSI acquisition parameters are described in [5,41]. In brief, horizontal, high-resolution, T2-weighted (T2w) MRIs (TR/TEeff = 4200/36 ms) were acquired using a RARE (Rapid Acquisition with Relaxation Enhancement) sequence (field of view (FOV), 19.2 × 19.2 mm; number of slices, 10; number of averages, 4). The T2w MRI resolution ranged from 75 × 75 to 150 × 150 μm^2^/pixel and the slice thickness ranged from 0.5 to 1 mm, depending on the case studied. The MRSI for this study was acquired at short TE (12–14 ms), using a 2D CSI (Chemical Shift Imaging) sequence with PRESS localisation, where: FOV, 17.6 × 17.6 mm; volume of interest (VOI), (5.5 × 5.5 × 1.0 mm) and with ST, 1 mm; TR, 2500 ms; SW, 4006.41 Hz; number of averages, 512. Water suppression was performed with VAPOR, using a 300 Hz bandwidth [5,41]. Short TE typically shows complex patterns, including metabolites with short and long T2 relaxation times, e.g., lipids, glutamine/glutamate and myoinositol, with an overall suitable signal-to-noise ratio. The MRSI data grid was formed by an array of 10 × 10 voxels, and the MR spectrum from each voxel contained 692 data points. This volume of interest was manually positioned approximately in the centre of the brain, based on the reference image, in a way that it would include most of the tumour mass and part of the normal/peritumoural brain parenchyma. MRI/MRSI data were always acquired prior to TMZ administration if they took place on the same day. In single-point cases (see Section 2.1.4), mice were euthanised at chosen time points for histopathological validation, as described in [5]. In longitudinal cases, mice were followed up periodically during a transient response until relapse and were euthanised at the endpoint. The primary outcome pursued with TMZ treatment is essentially the increase in the survival rate. However, the intermediate endpoint biomarkers as surrogates for the primary outcome were tumour volume changes (in the longitudinal explored cases and Ki67 immunostaining in single-point cases where animals were euthanised before the endpoint (e.g., to assess proliferation by Ki67 immunostaining)).

#### 2.1.3. MR Data Processing and Post-Processing

MRI T2w high-resolution images were used for the tumour volume calculation. MRSI data were initially pre-processed at the MR workstation with ParaVision 5.0 (Bruker BioSpin) and later post-processed with 3DiCSI v1.9.10 [42] and exported in ASCII format. The total number of points of each acquired voxel (original data) is 2048, distributed over 13.3 ppm. However, in both previous and current work, the authors have focused on the spectral window that concentrates the most relevant metabolites, i.e., between 0 and 4.5 ppm, which also avoids the residual water signal observed at 4.75 ppm. This finally resulted in 692 data points being analysed. The Dynamic MRSI Processing Module (DMPM) [43] running over MATLAB (MathWorks, Natick, MA, USA) was used to align and normalise all the spectra to unit length (UL2) [44].

#### 2.1.4. Single-Point and Longitudinal Cases Used

This study analysed a total of 28 mice, of which 21 were single-point cases and 7 were longitudinal. The single-point cases included a control (untreated) group of 7 mice (i.e., C32, C69, C71, C179, C233, C234, C278) previously studied in [17] and another group of 14 cases previously studied in [5,7], of which 8 were treated with TMZ, as described in Section 2.1.1 (i.e., C415, C418, C437, C525, C527, C575, C586, C584), and 6 were additional control, untreated cases (i.e., C255, C288, C351, C520, C529, C583). From the treated cases, C575 and C584 received three cycles of TMZ prior to euthanisation, whilst the rest of the group received two cycles. The longitudinal cases, recorded in Table 1, received three cycles of TMZ and were followed up for several days. Most of these cases were previously described in [5].

#### 2.1.5. Data Used for the Development of the Models

The voxels from each MRSI grid were labelled by experts as normal/non-tumour (N), tumour in response to therapy (R), control/untreated tumour (T) or unknown (usually peritumoural areas or edges of the grid). In order to assign the ‘R’ label, in longitudinal cases, we have considered the time points at which tumours present transient growth arrest or a volume decrease, consistent with Stable Disease or Partial Response according to our modified RECIST criteria described in [5]. The voxels labelled as N, R and T from selected MRSI grids were used to develop the training and test sets, as shown in Table 2. 

For the development of the models, we randomly selected 21 of the cases for training, which resulted in 83 MRSI grids. The remaining seven cases, i.e., C32, C179, C278 (single-point control cases), C437, C525, C584 (single-point treated cases) and C817 (longitudinal, treated), were reserved as an independent test set, resulting in 30 MRSI grids. Therefore, the split of cases was 75% and 25% for the training and test sets, respectively.

### 2.2. Model Development

#### 2.2.1. Hierarchical Classification Approach

**Figure 1:**
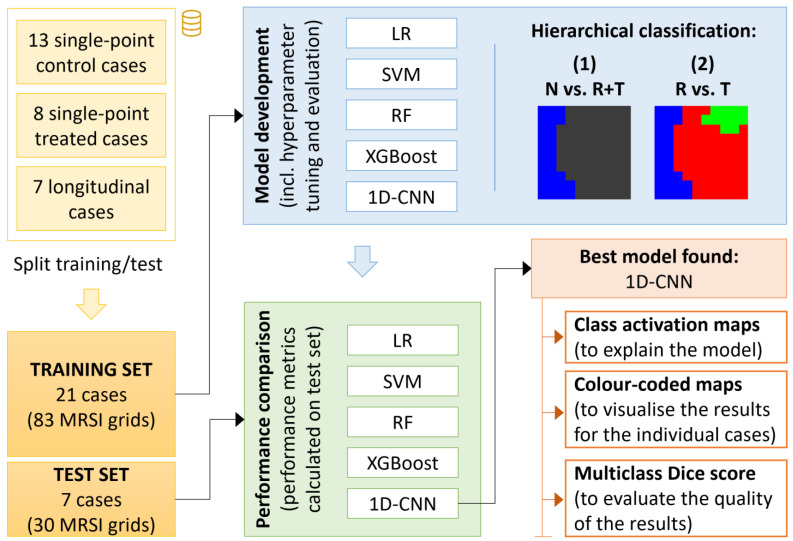
Summary of the study design.

We performed a hierarchical classification approach in which we first trained a classifier to discriminate between the normal brain parenchyma (N) and the tumour regions, regardless of whether they received treatment or not (i.e., including voxels labelled as R or T), which will be referred to as [N vs. R + T]; then, we trained a second classifier to distinguish between the areas of the tumour in response to TMZ (R) from those that were either control/untreated tumours (T), which will be referred to as [R vs. T]. The decision of using a hierarchical classification approach (i.e., N vs. R + T first, and then R vs. T) rather than modelling a multi-class classifier (i.e., R vs. T vs. N) was to facilitate the models to learn the differences between classes R and T due to the similarities between these two groups. A summary of the proposed methodology can be found in Figure 1.

#### 2.2.2. Methods Used for Classification

For the implementation of the classifiers, we proposed the use of CNN [40], specifically, 1D-CNN, which we benchmarked against four other methods, i.e., logistic regression (LR) [45], support vector machines (SVM) [46], random forest (RF) [47] and extreme gradient boosting (XGBoost) [48]. These are widely used machine learning algorithms with distinct characteristics.

LR is a statistical modelling technique used to predict categorical outcomes by estimating the probability of an event occurring based on a set of independent variables. It assumes a linear relationship between the predictors and the log odds of the outcome and uses a logistic function to transform these log odds into probabilities. 

SVM seeks to separate data into distinct classes by finding an optimal hyperplane in a high-dimensional feature space. It aims to maximise the margin between different classes while minimising the classification error and can handle both linearly separable and non-linearly separable data by using kernel functions to implicitly map the input data into a higher-dimensional space. 

RF is an ensemble learning method that constructs several decision trees and combines their predictions to make accurate classifications. Each tree in the forest is built using a random subset of the training data and random subsets of the predictor variables, resulting in a diverse set of trees that collectively provide robust and reliable predictions. 

XGBoost is also an ensemble learning technique that combines the outputs of multiple weak prediction models, typically decision trees, to create a powerful and accurate model. XGBoost employs a gradient boosting framework, where each subsequent tree is built to correct the mistakes made by the previous trees. It optimises a specific loss function by iteratively fitting new trees to the residuals of the previous predictions, resulting in a highly flexible and effective predictive model.

CNNs are DL [49] architectures that specialise in processing and extracting features from input data with a grid-like structure (e.g., images). One-dimensional CNNs (1D-CNNs) can be employed for time series and sequence analyses [50] by sliding a convolutional window across the sequence, extracting local features and leveraging the learned filters to capture temporal patterns and make predictions, making them particularly useful for the analysis of metabolomic datasets using whole pattern inputs.

Each method has its strengths. LR is interpretable and computationally efficient, but it is limited, as it is a linear classification algorithm. SVM is powerful in high-dimensional spaces and can handle both linear and nonlinear relationships. RF and XGBoost are known for their strong predictive capabilities, handling complex interactions and dealing with high-dimensional datasets. However, RF tends to be more computationally intensive due to its ensemble nature, whereas XGBoost efficiently handles large datasets by parallelising the boosting process. CNNs excel in image and sequence analysis, leveraging hierarchical feature extraction and parameter sharing, yet they demand substantial computational resources. The choice of algorithm depends on the specific problem and requirements.

#### 2.2.3. Hyperparameter Tuning

Hyperparameter tuning was performed to optimise the configuration of the parameters of the machine learning models. The aim was the identification of the optimal settings (which are not learned from the data but are set by the user before the training process) that maximise model performance [51].

In the case of the 1D-CNN, we optimised various hyperparameters that govern the architecture and training of the network. These included the number of convolutional layers (two to four layers), with the number and size of filters (from 16 to 144, with a step size of 32) and kernel size (from 3 to 12, with a step size of 3). We also optimised the number of units of the dense layer (from 64 to 512 units, with steps of 64) and the learning rate (from 10^−4^ to 10^−2^) of the Adam optimiser. LeakyReLU was chosen as the activation function of the convolutional and dense layers, with He normal as the kernel initialiser.

The algorithms used for comparison, i.e., LR, SVM, RF and XGBoost, were also optimised to avoid an unfair comparison with the deep learning model. For this, we performed a random search of the optimal values. In the case of LR, we searched for the optimal regularisation strength (best ‘C’ found: 545.6), penalty type (best ‘penalty’: ‘elasticnet’) and solver (best ‘solver’ found: ‘saga’). For SVM, we included the regularisation parameter (best ‘C’ found: 10) and the kernel function (best found was ‘rbf’: radial basis function or Gaussian kernel, with a gamma of 0.5). For RF, we searched for the optimal number of trees in the forest (best ‘n_estimators’ found: 1000) and the maximum depth of each tree (best ‘max_depth’ found: 40). In the case of XGBoost, we included the number of boosting stages or decision trees to be built (best ‘n_estimators’ found: 100), the learning rate (best ‘learning_rate’ found: 0.1), the fraction of features to consider when building each tree (best ‘colsample_bytree’ found: 0.75) and the maximum depth of individual decision trees in the ensemble (best ‘max_depth’ found: 12). In addition, the same splits for training and testing were used for training and evaluating the models developed with the different algorithms.

### 2.3. Model Explainability

#### 2.3.1. Class Activation Maps

To provide insight into the decision-making process of the 1D-CNN model, we used Grad-CAM, short for gradient-weighted class activation mapping [52]. Grad-CAM utilises the gradients of a target class (the class the network predicts) with respect to the final convolutional layer of a deep neural network. These gradients indicate the sensitivity of the predicted class to changes in the activation values of the convolutional layer. By weighting the activations of the final convolutional layer based on these gradients, Grad-CAM generates a class activation map that highlights the regions of the input data (the MRSI spectra in this study) that were crucial for the network prediction. It provides a form of visual explanation that aids in understanding and validating the model’s decision-making process.

#### 2.3.2. Colour-Coded Maps

Colour-coded maps were produced based on the class assignment by the two models. For this, the [N vs. R + T] model is applied to all the spectra (voxels) for any given MRSI grid. The voxels that are predicted as normal (N) are assigned a blue colour. Then, the [R vs. T] model is applied to all the remaining voxels (those that were not predicted as N). All the voxels from this ‘not N’ group that are predicted as T by the second model are then assigned a red colour, and those predicted as R are assigned green. In this way, every voxel from the MRSI grid will be assigned a colour: blue for N, red for T and green for R. These colour-coded maps are a form of a nosologic image of the brain [53], which summarises the presence of different tissue types in a single image by colour-coding each voxel according to the class it is assigned to.

#### 2.3.3. Evaluation of the Colour-Coded Maps Using a Dice Score

The Dice score [54], also known as the Dice coefficient or Dice similarity coefficient, is a metric commonly used to evaluate the similarity between two masks, as it quantifies the spatial overlap between them and provides a measure of their agreement. The Dice score is calculated by dividing twice the intersection of the predicted and ground truth masks by the sum of their individual pixel or voxel counts. It ranges from 0 to 1, where a score of 1 indicates a perfect match between the images, and a score of 0 indicates no overlap. For the cases of multiple classes, the Dice score can be extended to calculate the score for each class individually and then average them to obtain the multiclass Dice score. The multiclass Dice score provides a good evaluation of the overall segmentation performance when dealing with multiple classes simultaneously.

In this study, we use the multiclass Dice score to quantitatively evaluate how much overlap there is between the expected ground truth and the generated colour-coded maps, i.e., how accurate the produced colour-coded maps are. For this, we use a selection of cases with the following criteria: (i) single-point cases followed by euthanisation and, when available, histopathological validation; (ii) for control cases, all available cases were used; (iii) for treated cases, we have selected cases that received at least two TMZ cycles and either presented tumour growth arrest, changes in the proliferation index or mitoses/field counting or both. From our experience, cases undergoing only the first TMZ cycle could show mixed results and therefore would not be appropriate for validation purposes. We calculate the multiclass Dice score using the predicted and ground truth masks at the voxel-level resolution (not at the pixel level). 

## 3. Results

### 3.1. Selection of the Best Models

#### 3.1.1. Training of the Models

For the training of the models, all the voxels from each individual subject were ensured to be uniquely assigned to either the training or the test set (25% of the cases were reserved and used as an independent test set). This is to avoid what is known in the machine learning literature as ‘data leakage’, which is when information from the test set or about the target variable leaks into the input of the model during the training. Due to class imbalance (see Table 2), we used Synthetic Minority Oversampling TEchnique (SMOTE) [55] on the training data and increased the size of the smaller class to match the size of the larger class.

#### 3.1.2. Comparison and Evaluation of the Different Algorithms

For the comparison of the classifiers produced by the different machine learning algorithms, several metrics were used to evaluate their performance, i.e., accuracy, sensitivity, specificity, precision and F1-score. These metrics were calculated based on the areas labelled by the experts as N, T or R, not on the whole MRSI grid (meaning that the areas labelled as unknown were not used). Therefore, these results should be used with caution and mainly for the purpose of comparing the different classifiers. The results are compiled in Table 3 and Table 4 for [N vs. R + T] and [R vs. T], respectively.

Accuracy, specificity and sensitivity show an indication of the correct classifications that were made out of all the classifications, the negative cases made out of the total number of original negative cases and the positive cases made out of the total number of original positive cases, respectively. Precision focuses on the quality of positive predictions, indicating how many of the predicted positive cases are actually correct. The F1-score is a metric that combines precision and sensitivity (also known as recall) into a single value to provide a balanced evaluation of a model’s performance. In the [N vs. R + T] model, the negative class is N (hence, R + T is the positive class), and in the [R vs. T] model, the negative class is T (hence, R is the positive class).

According to the results in Table 3 and Table 4, 1D-CNN consistently outperformed the rest of the methods. Therefore, we will focus our attention on the 1D-CNN models for the rest of this study. 

#### 3.1.3. Description of the Best-Performing Models

As detailed in Section 3.1.2, the best-performing classifiers were developed using 1D-CNNs. These models were produced after optimising their hyperparameter to ensure the best learning and generalisation capabilities of the models. The details of their architectures can be found in Table 5 and Table 6. The optimal learning rate for the Adam optimiser used was 0.00081 for the [N vs. R + T] model and 0.00075 for the [R vs. T] model.

### 3.2. Model Explainability

#### 3.2.1. Class Activation Maps

**Figure 2:**
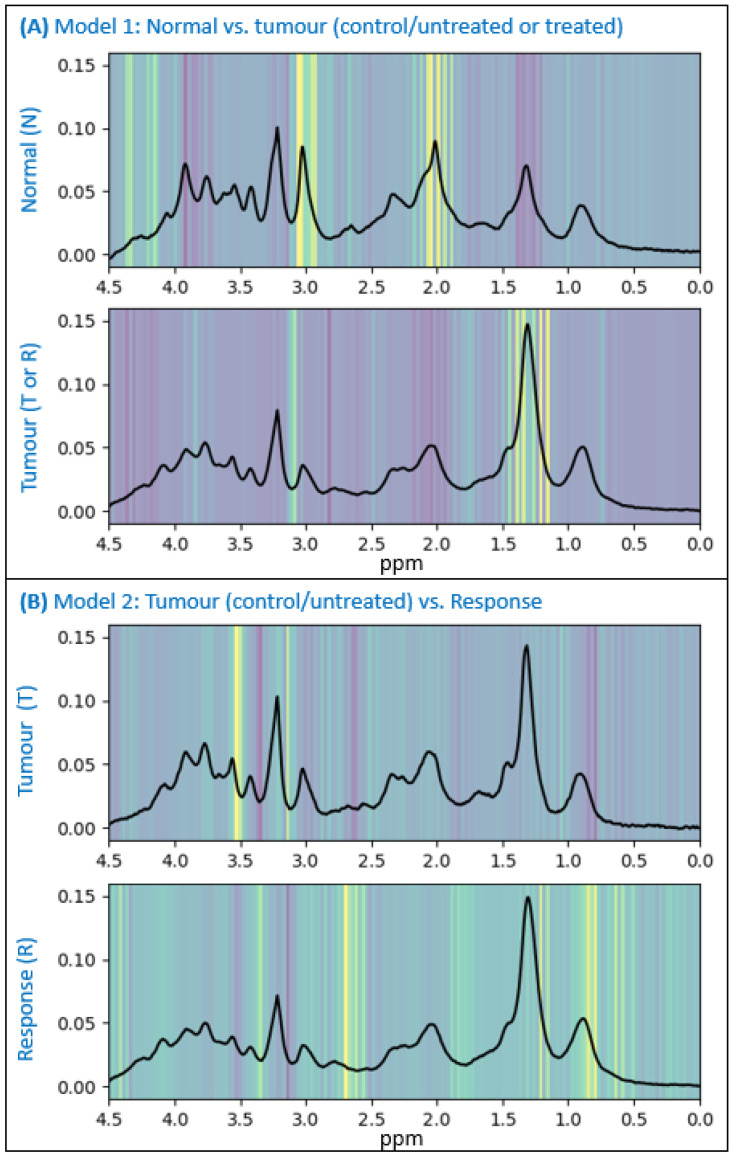
Activation maps of the class averages. They show the areas of interest to the models for classification. (**A**) Activation maps for the [N vs. R + T] model. (**B**) Activation maps for the [R vs. T] model. Class averages represent the average of the spectra (voxels) used for training the 1D-CNN models.

We applied Grad-CAM to the output of our two selected 1D-CNN models and overlaid the activation maps generated on the average of the classes, as shown in Figure 2. They explain, in a visual manner, the decisions made by the respective models. The higher values, indicated by the intensity of the yellow areas (note that they use a viridis colour palette), show the most influential areas and parts of the spectra that the models are taking into account for the separation of the classes.

#### 3.2.2. Colour-Coded Maps

**Figure 3:**
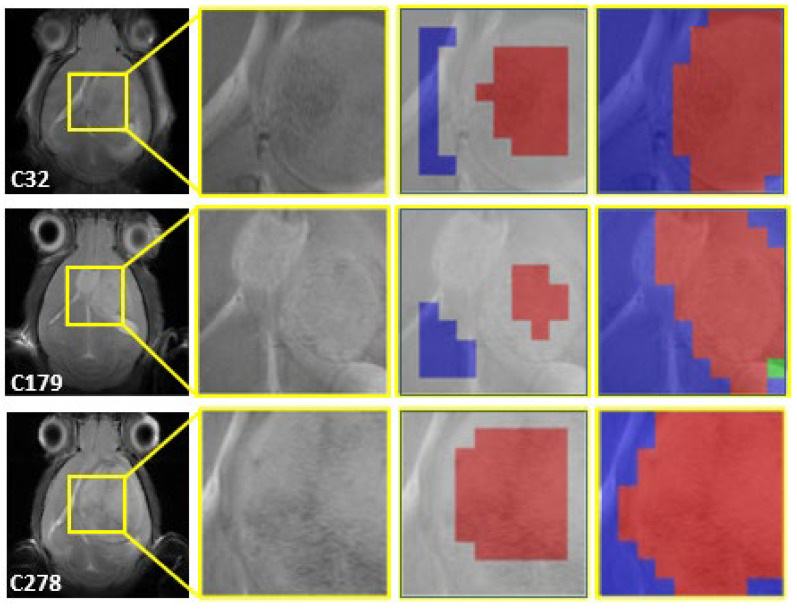
Single-point control cases. Three representative cases are shown. First column, horizontal T2w MRI with the MRSI grid position shown (yellow square). Second column, enlarged view of the tumour/peritumoural zone used in MRSI acquisition. Third column, voxels labelled as N (blue) or T (red) by the experts, superimposed to the corresponding MRI zone. Fourth column, the resulting coloured image generated (see Section 2.3.2), superimposed to the corresponding MRI zone.

**Figure 4:**
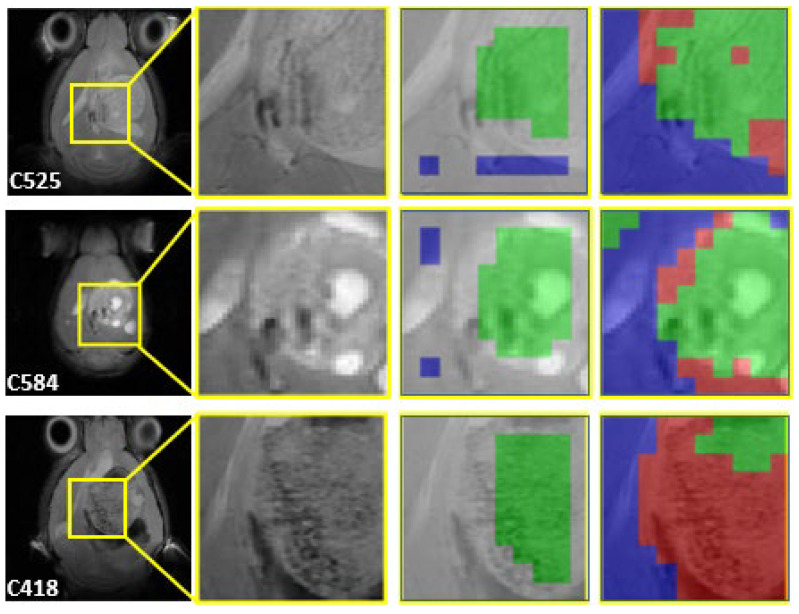
Single-point treated cases. Three representative cases are shown. First column, horizontal T2w MRI with the MRSI grid position shown (yellow square). Second column, enlarged view of the tumour/peritumoural zone used in MRSI acquisition. Third column, voxels labelled as N (blue) or R (green) by the experts, superimposed to the corresponding MRI zone. Fourth column, the resulting coloured image generated (see Section 2.3.2), superimposed to the corresponding MRI zone.

**Figure 5:**
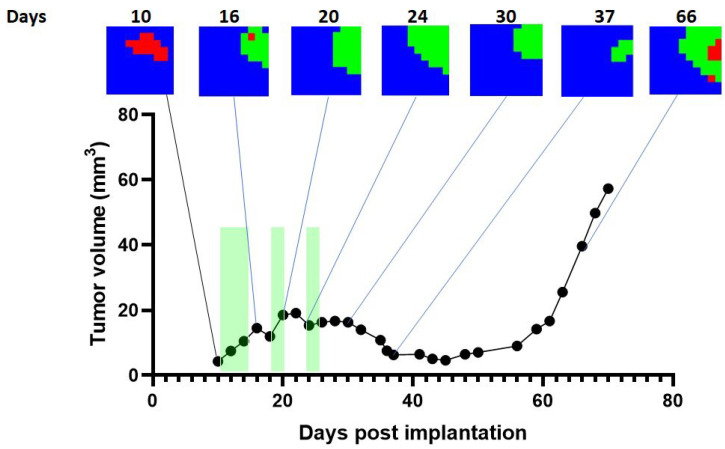
Representative longitudinal case C817. Each point represents a T2w/MRSI exploration. The *y*-axis shows the tumour volume, while the *x*-axis represents the day after tumour implantation. Green bars indicate the TMZ administration periods. This tumour started to show a volumetric response after the first TMZ cycle, about day 18 post-implantation (p.i.). The response was sustained, even showing a tumour volume decrease after day 30 p.i. After day 56 p.i., the tumour started to regrow, finally relapsing with exponential growth. The selected coloured images (Section 2.3.2) illustrate the evolution observed in this case, which presented a significant survival enlargement, since untreated mice have an average survival of 21 days.

Representative cases are shown in Figure 3 and Figure 4 for single-point cases and in Figure 5 for a longitudinal case, at different days post-implantation (p.i.). Details about the chosen cases are explained in Table 7. 

#### 3.2.3. Evaluation of the Colour-Coded Maps

The evaluation of the generated colour-coded maps was determined using the Dice score, which shows the overlap between the produced colour-coded maps and the expected ground truth. The Dice score was calculated individually for each of the expected areas in each case, i.e., normal (N), control/unresponsive (T) and treated/responding (R), to ensure enough evidence was provided to assess the quality of the models. In addition, we calculated the Dice scores of the results obtained when using the semi-supervised approach using sources from [5] for a comparison of the two modelling approaches on the same cases. These results are compiled in Table 8.

## 4. Discussion

Although deep learning models have demonstrated strong classification performances in many scenarios and fields, they are not fully utilised in tackling healthcare problems due to their black-box nature, which leads to a lack of trust [56,57]. This is exacerbated when it comes to decisions impacting patient management and treatments.

In the case of malignant brain tumour patients, decisions about treatment management are of paramount importance and rely mostly on non-invasive approaches, especially MRI. Spectroscopic approaches such as MRSI have been proven to have an added value reflecting some local changes earlier than anatomical changes related to a volumetric decrease, but it is currently underused in clinical settings. Challenges in data handling, processing and interpretation, in addition to the lack of standardisation, can at least partially explain why it is fully unexploited. Most authors employing spectroscopic approaches for brain tumour management investigate few signals or signal ratios. Moreover, clinicians are more used to imaging-like outputs and less familiarised with spectroscopic features. Therefore, machine learning approaches that could be turned into imaging-based outputs could have much better acceptance, provided they account for enough explainability regarding the decision pathways and also have suitable biological validation.

For evident ethical reasons, it is not feasible to perform studies with repeated MR explorations in clinical patients, and it is not acceptable to perform repeated biopsies for biological validation. Thus, preclinical studies might be of relevance in this landscape, allowing for (i) repeated and periodic MR explorations, (ii) the establishment of a control, non-treated group and (iii) the euthanisation of chosen mice at defined time points in order to validate the spectroscopic findings. Our group has a large track dealing with preclinical brain tumour models, their treatment and non-invasive therapy response follow-up [9,38,39,58], with a special view to the immunocompetent GL261 GB model, which recapitulates some features of human GB and presents transient or sustained responses under TMZ treatment. 

Moreover, the strong contribution of the host immune system in response to therapy cannot be neglected (e.g., [59]), and, in this sense, the use of immunocompetent models such as GL261 is preferred over the use of immunosuppressed ones. Thus, we wanted to explore whether we could improve our previous work, describing source-based classification for investigating the response to TMZ treatment in such preclinical GB model [5]. 

The GL261 GB model has an average survival of 21 days when it is not treated. Such survival proved to be increased to 34 days with the 5-2-2 treatment protocol [7] and to even higher values with some protocol adaptations [9], proving a consistent response. In this work, we used well-defined datasets for training the new models using machine learning. The first step of the hierarchical classification approach goes toward the straightforward discrimination between affected and non-affected tissue. The second step deals with much less obvious changes, which are difficult to detect even by an experienced spectroscopist, and the local heterogeneity of GB in response might also be considered, in which tumours can have responding and non-responding mixed zones. This classification can be further complicated since even untreated (or unresponsive) tumours can deal with a non-negligible amount of natural cell death (i.e., not produced by a therapeutic agent), which in turn would lead to local biochemical and molecular changes spotted by the classifier.

In this study, we implemented and evaluated several machine learning approaches, ranging from linear models to more sophisticated deep learning architectures using 1D-CNN. Table 3 and Table 4 show the performance of all of these models and how 1D-CNN outperformed the rest. These results were expected, as 1D-CNNs can outperform other linear and non-linear models due to their ability to capture local patterns and features within sequential data, making them well suited for this study. Their parameter-sharing technique reduces overfitting and enhances efficiency. Additionally, their hierarchical representation learning enables the extraction of abstract features at different levels, making them highly effective in capturing complex relationships in the data.

The representative cases using the 1D-CNN models shown in Figure 3 and Figure 4 prove that the superimposition of the colour-coded images over the MRI anatomical tumour mass is excellent, even in cases with atypical growth such as C179 in Figure 3. 

The activation maps shown in Figure 2 provide model explainability, as they offer an intuitive way to understand the developed 1D-CNN models by highlighting the regions of the input MR spectra that are most influential in the models’ decision-making process. This helps us to gain insights into the models’ reasoning and builds trust by providing interpretable explanations for the models’ predictions. Figure 2 (top) for the [N vs. R + T] model indicates that the signals most contributing to the classification decision are 3.04 ppm (consistent with a creatine signal), 2.93 ppm (contributed by creatine and glutathione), 2.00–2.04 ppm (consistent with an N-acetyl aspartate- or N-acetyl-containing compounds signal) and 1.34 ppm and other near positions such as 1.21 and 1.4 ppm (consistent with a mobile lipids/lactate signal). This is coherent with our previously registered patterns in the same preclinical model [44], also described by others [60].

Both creatine and N-acetyl aspartate are typical metabolites from normal/non-affected brain tissue, being an energy level indicator and a neuronal marker, respectively, while high signals of mobile lipids/lactate are usually observed in malignant tumours/tissues [61], related to membrane turnover and tumour metabolism, respectively. Such differences are also described for human GB [62,63,64], reinforcing both the suitability of the signals chosen and the applicability of a preclinical developed model to a clinical setting. It is also worth noting that these changes are easily spotted by a trained spectroscopist, as opposed to changes challenged by the second step of the hierarchical classification approach. 

Regarding glutathione, it is not one of the majority signals, being less represented in the literature of non-invasive brain tumour studies. It is the most abundant non-enzymatic antioxidant in mammalian cells, playing a crucial role in regulating tumour oxidative stress. GB might present elevated glutathione levels, especially under hypoxic conditions, and this metabolite can also play a role in resistance to therapy [65].

The cases for classifier training were carefully chosen, and some representative/interesting results are shown in Figure 4. In this figure, we can see two cases showing mostly a responsive pattern around the investigated tumour zones (C525 and C584), while a third case shows a heterogeneous feature, with a small responding zone surrounded by tumour tissue characterised as control/unresponsive (C418). This result is in agreement with C418, having a large tumour volume, which did not show growth arrest after the first TMZ cycle, with a higher number of mitoses/field in comparison with the other two, combined with a Ki67 value comparable to those of untreated cases. Still, the same trend was found for case C418 in our previous study [5]. 

On the other hand, C525 and C584 did show a lower number of mitoses/field and volume values in the range of or below the average of responding cases. Overall, it suggests that the classifier can learn and handle small local differences, correctly spotting heterogeneous tumours. Regarding the activation maps, Figure 2 (bottom) for the [R vs. T] model shows that decisions are driven by the 3.54 ppm signal (corresponding to the myo-inositol/glycine position, not resolved at this magnetic field) and signals compatible with mobile lipids/lactate signals (1.23, 0.8 and 0.83 ppm), although not over the described maximum positions. 

The mobile lipids/lactate signal was also one of the differential trends between the responding and control/unresponsive zones described by us [5] in this same preclinical GB model. Lactate is described to be an indicator of altered tumour metabolism, with a less efficient process of aerobic glycolysis that converts pyruvate into Lac, ultimately resulting in energy (ATP) [66,67], and it has been investigated for a long time as one of the signals for assessing the response to therapy in human gliomas [68]. The appearance of mobile lipids due to cell apoptosis, especially from methylene and methyl groups at ca. 1.3 and 0.9 ppm, has been described by several authors (e.g., [69,70]. Myo-inositol is involved in central nervous system osmoregulation and is elevated in response to brain inflammation, highlighting its potential in assessing the response to therapy [67]. Its concentration may indicate a metabolic reaction to osmotic changes in the brain, and some authors have investigated the potential of myo-inositol to assess the response to antiangiogenic therapy [71].

Finally, our results suggest that one additional spectral position contributing to this classification is at 2.71 ppm. This could also have a biochemical explanation: some authors have described the appearance of a ca. 2.8 ppm signal from polyunsaturated fatty acids (PUFA) appearing due to cell apoptosis after treatment [72], and it was also one of the differential signals from untreated vs. treated–responding in our previous work [5]. Since this signal has a broad feature in vivo (in our datasets, when present, it comprises the 2.7 to 2.9 ppm zone), we cannot discard that this feature is spotting the rise in PUFA signals due to the cell death triggered by treatment. Other minor signals in activation maps lack evident biochemical signification, and their meanings are unclear.

It is also worth mentioning that TMZ treatment with this tumour model induced the appearance of giant, multinucleated cells [8], a well-described effect of TMZ also over human GB cells [73]. These polyploid cells usually have senescent characteristics and a higher metabolic rate than the euploid control population [74]. Last but not least, we should also bear in mind that MRSI acquisitions are a reflection not only of tumour cells but of the whole tumour microenvironment. Tumour-associated macrophages may constitute up to 40% of the tumour mass [75], displaying different phenotype characteristics, and the MRSI pattern will also be related to their metabolism. Our previous work with GL261 GB under TMZ treatment proved that tumours with a clear response to treatment showed a relevant change in macrophage phenotypes, switching from a more ‘antitumour’ phenotype [76], and it was already described by others that different macrophage phenotypes will have different metabolic profiles [77], including glycolytic pathways involving lactate production. Additionally, immune cells can also display relevant changes in their lipid profiles, as reviewed by [69]. 

The method was also applied to our longitudinal cases. Accordingly, Figure 5 shows the application of the proposed 1D-CNN classifier to one characteristic case. In this case, the exploration pre-treatment shows a control/unresponsive classification, which turns into responding in subsequent days with growth arrest and a tumour volume decrease. At the relapsing time frame, a heterogeneous classification arises, suggesting the reappearance of proliferating and/or unresponsive zones.

When evaluating the colour-coded maps generated by our proposed approach against the ground truth, we could see that our method was overall very good at identifying the three areas (Table 8). It was particularly good at identifying the response signal (with an average overlap of 92%), followed by the control/unresponsive tumour region (with 90%). On some occasions, voxels were identified as T by the system when it was expected to be R (according to the pre-defined ground truth). This is not surprising, since the heterogeneity of GB is well recognised in the literature both at preclinical and clinical levels [78,79]. In other words, it is perfectly feasible that a tumour is heterogeneously formed by responding and unresponsive/highly proliferative zones, with a net result of an overall response in the tumour volume. Still, we preferred to fall on the conservative side for the sake of the comparisons and record those voxels as incorrectly classified, in accordance with the pre-defined ground truth.

Utilising the same evaluation process with the Dice score, we also compared the proposed new method with our previously developed semi-supervised approach using NMF [5]. The comparison yielded interesting insights—particularly, how much more accurate the 1D-CNN approach is at identifying the responding and the control/unresponsive tumour regions, whilst also managing to outperform the normal region.

## 5. Conclusions

In this paper, we propose a new methodological approach using 1D-CNN for therapy response assessment in the GL261 GB preclinical model under TMZ treatment. The 1D-CNN models developed were compared against ground truth and against other previous methods, providing evidence of high performance and competitiveness. Model explainability was also achieved via the development of Grad-CAM activation maps, which highlighted the areas of importance for model prediction, enabling further understanding and trustability in the results. The proposed methodology may provide new and better opportunities for therapy response assessment, with capabilities of detecting even heterogeneity within responding tumours. This might provide a potential pipeline for therapy response tracking, also bearing the potential of producing earlier relapsing hints.

## Figures and Tables

**Table 1:** Longitudinal cases. Number of MRSI grids available and the days on which they were acquired (counting from when tumour cells were inoculated) per mouse.

Mice:	Number of MRSI Grids:	Days When They Were Acquired:
C797	6	10, 15, 17, 18, 20, 22
C806	8	11, 12, 14, 16, 18, 20, 22, 24
C808	11	10, 12, 14, 16, 18, 20, 22, 24, 26, 28, 33
C809	12	11, 12, 14, 16, 18, 20, 22, 24, 26, 28, 31, 33
C817	24	10, 12, 14, 16, 18, 20, 22, 24, 26, 28, 30, 32, 35, 39, 45, 48, 50, 56, 58, 61, 63, 66, 68, 70
C819	18	10, 12, 14, 16, 18, 20, 22, 24, 26, 28, 30, 32, 34, 36, 39, 41, 43, 45
C821	13	10, 12, 14, 16, 18, 20, 22, 24, 26, 28, 30, 32, 34

**Table 2:** Labelled voxels used for analysis, including the number and percentage of records per class.

Class (Labelled Voxels)	Number of Records	Percentage of Records
Normal/non-tumour (N)	998	39.5%
Tumour in response (R)	1151	45.6%
Control/untreated tumour (T)	376	14.9%

**Table 3:** Performance of the [N vs. R + T] model on the labelled voxels of the independent test set.

	Accuracy	Sensitivity	Specificity	Precision	F1-Score
LR	96.538%	96.157%	97.878%	99.377%	97.010%
RF	97.711%	97.664%	97.878%	99.387%	97.771%
SVM	97.946%	97.664%	98.939%	99.692%	98.297%
XGBoost	96.890%	96.534%	98.143%	99.457%	97.332%
1D-CNN	99.328%	99.344%	99.293%	99.671%	99.319%

**Table 4:** Performance of the [R vs. T] model on the labelled voxels of the independent test set.

	Accuracy	Sensitivity	Specificity	Precision	F1-Score
LR	91.560%	92.674%	85.052%	97.312%	88.699%
RF	92.238%	92.851%	88.660%	97.952%	90.707%
SVM	84.476%	83.230%	91.753%	98.332%	87.284%
XGBoost	88.621%	88.976%	86.598%	97.486%	87.767%
1D-CNN	99.750%	99.810%	99.310%	99.905%	99.560%

**Table 5:** Architecture of the best-performing 1D-CNN model for [N vs. R + T].

Layers	Output Size	Parameters
1D Convolution	692	Filters: 80, Kernel size: 12
1D Max Pooling	346	Pool size: 2
1D Convolution	346	Filters: 112, Kernel size: 9
1D Max Pooling	173	Pool size: 2
1D Convolution	173	Filters: 112, Kernel size: 3
1D Max Pooling	86	Pool size: 2
Flatten	9632	
Dropout	9632	0.5
Dense	192	Units: 192
Dropout	192	0.5
Dense	1	Units: 1

**Table 6:** Architecture of the best-performing 1D-CNN model for [R vs. T].

Layers	Output Size	Parameters
1D Convolution	692	Filters: 144, Kernel size: 9
1D Max Pooling	346	Pool size: 2
1D Convolution	346	Filters: 48, Kernel size: 6
1D Max Pooling	173	Pool size: 2
1D Convolution	173	Filters: 112, Kernel size: 3
1D Max Pooling	86	Pool size: 2
Flatten	9632	
Dropout	9632	0.5
Dense	448	Units: 448
Dropout	448	0.5
Dense	1	Units: 1

**Table 7:** Data from tumour-bearing mice shown in Figure 3 and Figure 4. All individuals were euthanised right after the last T2w/MRSI acquisition, at the indicated post-implantation (p.i.) day, for histopathological validation.

Condition	Day p.i. (Range)	TMZ Cycles	Tumour Volume (Average ± SD mm^3^)	Ki67 Index (Average ± SD %) *	Mitosis/Field (Average ± SD) *
Control	16–19	-	93 ± 60	56.4 ± 1.9	13.9 ± 2.7
Treated	22–26	2–3	105 ± 48	49.3 ± 5.9	3.1 ± 1.0

* Ki67 immunostaining and mitoses/field counting were performed as described in [5] by histopathology experts. The final number of fields examined depended on the tumour size. Ki67 is a marker for the tumour proliferative status, which is expected to decrease under successful treatment. In the same line, the number of mitoses is expected to decrease in treated-responding cases. SD = standard deviation.

**Table 8:** Evaluation of the colour-coded maps. Dice score results for the normal (N), control/unresponsive (T) and treated/responding (R) groups. Results include the semi-supervised approach using sources from [5] and the proposed methodology using 1D-CNN. CI: Confidence Interval. Best values per group are highlighted in bold.

		Semi-Supervised NMF [5]			1D-CNN		
Mice	Condition	N	T	R	N	T	R
C32	Control	0.88	0.90	-	**0.91**	**0.93**	-
C69	Control	0.84	0.72	-	**0.95**	**0.91**	-
C71	Control	0.67	0.71	-	0.77	0.86	-
C179	Control	0.73	0.55	-	**0.86**	**0.89**	-
C233	Control	0.89	0.91	-	**0.92**	**0.94**	-
C234	Control	0.91	**0.94**	-	0.89	**0.94**	-
C278	Control	**0.90**	**0.87**	-	0.70	0.86	-
C255	Control	0.85	0.80	-	**0.86**	**0.88**	-
C288	Control	0.82	0.72	-	**0.93**	**0.86**	-
C351	Control	**0.86**	0.55	-	**0.86**	**0.87**	-
C520	Control	**0.68**	**0.90**	-	0.60	0.88	-
C529	Control	0.69	0.74	-	**0.86**	**0.96**	-
C583	Control	0.86	0.93	-	**0.94**	**0.96**	-
C437	Treated	0.76	-	**0.95**	**0.96**	-	0.92
C525	Treated	0.90	-	0.00	**1.00**	-	**0.87**
C527	Treated	0.94	-	0.79	**0.95**	-	**0.96**
C575	Treated	**0.96**	-	0.12	0.91	-	**0.91**
C584	Treated	0.94	-	0.69	**0.95**	-	**0.89**
C586	Treated	0.85	-	0.95	**0.90**	-	**0.99**
**Average** ± **CI:**		0.84 ± 0.04	0.79 ± 0.07	0.58 ± 0.31	**0.88 ± 0.04**	**0.90 ± 0.02**	**0.92 ± 0.03**

## Data Availability

A relevant set of control cases (MRI and MRSI data) is already available at the UAB Digital Repository (https://ddd.uab.cat/record/201551?ln=ca, last accessed on 4 August 2023). Additional cases will be made available upon reasonable request to the corresponding authors.
